# Medical Genetics, Genomics and Bioinformatics—2022

**DOI:** 10.3390/ijms24108968

**Published:** 2023-05-18

**Authors:** Vadim V. Klimontov, Konstantin A. Koshechkin, Nina G. Orlova, Marina I. Sekacheva, Yuriy L. Orlov

**Affiliations:** 1 Research Institute of Clinical and Experimental Lymphology—Branch of the Institute of Cytology and Genetics, Siberian Branch of Russian Academy of Sciences (RICEL—Branch of IC&G SB RAS), 630060 Novosibirsk, Russia; klimontov@mail.ru; 2 The Digital Health Institute, I.M. Sechenov First Moscow State Medical University of the Ministry of Health of the Russian Federation (Sechenov University), 119991 Moscow, Russia; 3 Department of Mathematics, Financial University under the Government of the Russian Federation, 125167 Moscow, Russia; 4 Institute of Personalized Oncology, I.M. Sechenov First Moscow State Medical University of the Ministry of Health of the Russian Federation (Sechenov University), 119991 Moscow, Russia; 5 Institute of Life Sciences and Biomedicine, Far Eastern Federal University, 690922 Vladivostok, Russia; 6 Agrarian and Technological Institute, Peoples’ Friendship University of Russia, 117198 Moscow, Russia

The analysis of molecular mechanisms of disease progression challenges the development of bioinformatics tools and omics data integration. We have presented an earlier series of journal special issues: “*Medical Genetics, Genomics and Bioinformatics”,* “*Medical Genetics, Genomics and Bioinformatics–2020*” and “*Medical Genetics, Genomics and Bioinformatics–2021*” [1]. This special journal issue “*Medical Genetics, Genomics and Bioinformatics—2022*” (https://www.mdpi.com/journal/ijms/special_issues/Medical_Genetics_2022, accessed on 12 May 2023) collected papers on medical genomics, human population genetics and computational biology applications in biomedicine, continuing the topic of medical genetics and genomics. Here, we focused on bioinformatics and systems biology approaches to medical genetics problems, molecular oncology and bioinformatics approaches for medical genomics.

The papers on bioinformatics applications were originally discussed at the “Bioinformatics of Genome Regulation and Structure/Systems Biology” (BGRS/SB) multiconference 2022 and its biomedical symposia in Novosibirsk, Russia (https://bgrssb.icgbio.ru/2022/, accessed on 12 May 2023). The current collection continues the series of post-conference journal special issues presenting the highlights from the set of meetings on genetics and systems biology highlighting recent trends in cancer genomics [2,3]. BGRS/SB discussions of systems biology and genomics achievements were published in thematic journal issues [3].

Molecular mechanisms of human disease progression [1,2] are being studied using omics technologies. We should acknowledge the current, parallel *IJMS* special journal issue, *Bioinformatics of Gene Regulations and Structure* (https://www.mdpi.com/journal/ijms/special_issues/Bioinformatics_Gene, accessed on 12 May 2023) and the new IJMS journal issue *New Sights into Bioinformatics of Gene Regulations and Structure* (https://www.mdpi.com/journal/ijms/special_issues/MVA479KFR7, accessed on 12 May 2023). The recent special issue on computational genomics in *Life* (https://www.mdpi.com/journal/life/special_issues/computational_genomics_life, accessed on 12 May 2023) and *Genes* journal also has works on medical bioinformatics [4] organized as a follow-up of BGRS/SB and special journal issue series.

This issue *Medical Genetics, Genomics and Bioinformatics—2022* presents recent studies on medical genomics. It contains eight research manuscripts and one review, each concerning a bioinformatics solution for the analysis of the molecular mechanisms underlying disease progression.

Jeong-An Gim [5] presented a review of Big Data analysis using a genomic information management system. The analysis of genomic information as Big Data can be applied for clinical and research purposes [6]. Tremendous volumes of genomic information are being generated, and the development of methods for its collection, cleansing, storing, indexing and serving must progress under legal regulation. The improvement of technology has enabled precision or personalized therapy based on genomic information, such as genotype, gene expression and DNA methylation patterns, solving challenging problems for application in the clinic.

Almost all the research papers in this special issue deal with NGS data to study disease mechanisms and find candidate genes. Ionut-Florin Iancu [7] analyzed aggregated genomic data as cohort-specific allelic frequencies for inherited retinal dystrophies (IRD). The authors built an allelic-frequency database for a heterogeneous cohort of genetic diseases to explore the aggregated genomic information in IRD in retrospective analysis with available clinical exome sequencing tests [8].

Elena Pudova et al. [9] analyzed transcriptomic data in prostate cancer. The authors performed RNA-Seq for experimental PC3 cell lines as well as for plasma exosome samples from patients with castration-resistant prostate cancer. The work by the same authors’ group on gene expression changes in prostate cancer following bioinformatic analysis [10] was published in the Research Topic on “Bioinformatics of Genome Expression” in the *Frontiers in genetics* special journal issue (https://www.frontiersin.org/research-topics/40408/bioinformatics-of-genome-regulation-and-systems-biology-volume-iii, accessed on 12 May 2023). Recent work by the same team on lymphatic dissemination in prostate cancer complemented this study [11]. Overall, E. Pudova and colleagues [11] have shown transcriptomic profiling of the experimental prostate cancer cell line, in the process of acquiring resistance to docetaxel, using the RNA-Seq approach.

Yaron Trink and co-authors studied heterogeneity in Wilms’ tumor pediatric malignancy related to faulty kidney development [12]. Wilms’ tumors are highly heterogeneous and contain varying proportions of cells [13]. Using a dataset of microarray gene expression measurements and an unsupervised machine learning algorithm, Y. Trink and colleagues have developed a computational classification model for Wilms’ tumors.

The topic of cell classification is continued by Olga Krasnova and colleagues [14] presenting regenerative medicine application on human pluripotent stem cells lines (embryonic stem cell line H9 and control human-induced pluripotent stem cells [15]). The authors completed a morphological assessment of growing colonies and cells that allow the best clones to be safely selected for further clinical applications. The morphological phenotype of each colony was classified using a visual analysis and associated with its potential for pluripotency and clonality maintenance. The authors have shown the fundamental possibility of constructing a morphological portrait of a colony that is informative for the automatic identification of the phenotype [14].

Mark Melamud et al. [16] studied cytokine profiles in autoimmune diseases, including systemic lupus erythematosus and multiple sclerosis. The authors reconstructed and analyzed the protein interaction network expanding the understanding of abnormal regulatory interactions in cytokine profiles associated with autoimmune diseases [17]. Autoimmune diseases were the aim of the next study by Andrey Shevtsov et al. [18]. Using single cell RNA-seq data for an in silico drug, the authors detected several promising small molecules to reverse the transcriptomic signatures of multiple sclerosis immune cells. Among these molecules, A. Shevtsov and colleagues also detected an FDA-approved multiple sclerosis drug, Mitoxantrone, supporting the reliability of the approach [18]. The single cell sequencing analysis is on trend for current cancer studies. In the parallel *IJMS* special issue, *Bioinformatics of Gene Regulations and Structure–2022* (https://www.mdpi.com/journal/ijms/special_issues/Bioinformatics_Gene, accessed on 12 May 2023) the same authors’ group presented a new tool for scRNA-seq imputation via integration with single-cell ATAC-seq that increases the power of the analysis [19].

Valeriia Danilchenko and colleagues [20] studied mutation in genes causing hearing loss. The authors analyzed known pathogenic variants across the SLC26A4 gene sequence presented in the Deafness Variation Database (https://deafnessvariationdatabase.org/, accessed on 12 May 2023) for the selection of potential diagnostically important parts of this gene. Initial diagnostic testing for hearing loss was suggested. Studying genetic variants in populations is an important approach applied earlier for diabetes [21]. Olga Saik and Vadim Klimontov [22] considered the challenging problem of COVID-19 complications and comorbidity including diabetes. Using text-mining-based approaches and the ANDSystem [23,24] as a bioinformatics tool, the authors reconstructed and matched networks related to hyperglycemia, diabetic complications, insulin resistance and beta cell dysfunction with networks of SARS-CoV-2-targeted proteins. The results expand the understanding of the molecular basis of diabetes and COVID-19 comorbidity [25,26].

Overall, the current special issue on bioinformatics confirmed research interests in medical genomics and bioinformatics studies [1,2]. We note here the new MDPI *IJMS* special issue *New Sights into Bioinformatics of Gene Regulations and Structure* topic at (https://www.mdpi.com/journal/ijms/special_issues/MVA479KFR7, accessed on 12 May 2023) to continue the paper selection on bioinformatics and genomics in human diseases, as well as the research topic at *Frontiers in genetics* (https://www.frontiersin.org/research-topics/53085/high-throughput-sequencing-based-investigation-of-chronic-disease-markers-and-mechanisms---volume-ii, accessed on 12 May 2023).
